# Cross-Modal Integration of Lexical-Semantic Features during Word Processing: Evidence from Oscillatory Dynamics during EEG

**DOI:** 10.1371/journal.pone.0101042

**Published:** 2014-07-09

**Authors:** Markus J. van Ackeren, Shirley-Ann Rueschemeyer

**Affiliations:** Department of Psychology, University of York, York, United Kingdom; Utrecht University, Netherlands

## Abstract

In recent years, numerous studies have provided converging evidence that word meaning is partially stored in modality-specific cortical networks. However, little is known about the mechanisms supporting the integration of this distributed semantic content into coherent conceptual representations. In the current study we aimed to address this issue by using EEG to look at the spatial and temporal dynamics of feature integration during word comprehension. Specifically, participants were presented with two modality-specific features (i.e., visual or auditory features such as *silver* and *loud*) and asked to verify whether these two features were compatible with a subsequently presented target word (e.g., *WHISTLE*). Each pair of features described properties from either the same modality (e.g., *silver, tiny*  =  visual features) or different modalities (e.g., *silver, loud*  =  visual, auditory). Behavioral and EEG data were collected. The results show that verifying features that are putatively represented in the same modality-specific network is faster than verifying features across modalities. At the neural level, integrating features across modalities induces sustained oscillatory activity around the theta range (4–6 Hz) in left anterior temporal lobe (ATL), a putative hub for integrating distributed semantic content. In addition, enhanced long-range network interactions in the theta range were seen between left ATL and a widespread cortical network. These results suggest that oscillatory dynamics in the theta range could be involved in integrating multimodal semantic content by creating transient functional networks linking distributed modality-specific networks and multimodal semantic hubs such as left ATL.

## Introduction

The embodied framework of language suggests that lexical-semantic knowledge (i.e., word meaning) is stored in part in modality-specific networks that are distributed across the cortex [1]–[4]. For example, words denoting colors (e.g., *red, green*) have been shown to engage parts of the ventral visual stream [5], while words denoting actions (e.g., *kick*, *pick*) engage the dorsal motor network [6]. In recent years, much has been done to understand the automaticity, flexibility and reliability of the link between action/perception and word meaning [5], [7]–[10]. The current study extends this body of literature by addressing the question of how distributed lexical-semantic features are *integrated* during word comprehension.

Although ample evidence for the link between word meaning and perception/action systems exists, the bulk of research in this field has reduced lexical-semantic information to one dominant modality (e.g., vision for *red* and action for *kick*). The motivation for focusing on single modalities is clearly methodological: by focusing on words with a clear association to one modality, good hypotheses can be generated for testing empirically. However, words clearly refer to items that are experienced through multiple modalities in the real world (e.g., a football is associated with both a specific visual form and a specific action), and embodied accounts of language have done little to address how multimodal information interacts during the processing of word meaning. The one exception to this rule has been the attempt to understand how lexical-semantic processing can be focused flexibly on information from one modality versus another. For example, van Dam and colleagues [10] demonstrated that words denoting objects that are strongly associated with both action and visual information (e.g., *tennis ball*) reliably activate both motor and visual pathways in the cortex. Interestingly, motor pathways also responded more strongly when participants were asked to indicate what to do with the object rather than what it looks like. Likewise, Hoenig and colleagues [8] have shown that even for objects with dominant modality-specific features (e.g., actions for artifacts), the pattern of activation in visual and motor networks is differentially modulated if a dominant (action) or non-dominant (visual) feature is primed. Notably, modality-specific networks show a stronger response to the target if the prime was not a dominant feature. Taken together, the studies by van Dam et al. [10] and Hoenig et al. [8] suggest that word meaning is partially stored in a network of areas that are recruited in a modality-specific and flexible way. However, it should also be pointed out that most of this evidence is of a correlational nature. As yet, little is known about the causal role of modality-specific networks in lexical-semantic processing, and how they are related to more abstract semantic knowledge [11], [12].

While studies highlighting the flexible recruitment of different types of modality-specific information confirm that single words are associated with multiple types of perceptual experience, it is still unknown how information from multiple sources in the brain (e.g., visual and action features) is united to form a coherent concept that is both visual and motoric. Cross-modal integration has been studied extensively with respect to object perception [13]–[16]. However, its role in forming lexical-semantic representations has been largely neglected, even within the embodied framework. Several theoretical perspectives have argued for the existence of amodal integration ‘hubs’ or foci, at which information relevant for lexical-semantic processing is combined [17], [18]. Neuropsychological data has provided compelling evidence that the anterior temporal lobes (ATL) may be a good candidate for such a hub [18], [19]. Thus, there is a general acceptance that information from distributed modality-specific networks is integrated in some way, somewhere in the brain. However, virtually no research has looked at what the neural mechanisms underlying semantic integration might be in these hub regions or more widely across the brain.

One way to investigate the mechanisms underlying integration across cortical areas is to study modulations in oscillatory power in EEG and MEG signals that have been related to network interactions at different cortical scales [20], [21]. Specifically, low frequency modulations (< 20 Hz) are often reported when tasks require the retrieval and integration of information from distant cortical sites, which is generally the case for memory and language [22]–[25]. In contrast, modulations in high frequency bands (>30 Hz) are observed when tasks require local, modality-specific, network interactions such as saccade planning or visual object binding [26], [27]. According to this framework, the specific network dynamics underlying the integrating of lexical-semantic features across different modalities should be reflected in a modulation in low frequencies.

The aim of the current study was to investigate what mechanisms underlie the integration of semantic features across modalities. This question was addressed in two experiments using a dual property verification task. Participants were asked to indicate whether a feature pair (e.g., *silver, loud*) is consistent with a target word (e.g., *WHISTLE*). Critically, the feature pair could either be from the same modality (e.g., both visual), or from different modalities (e.g., visual and auditory). In Experiment 1 we analyzed verification times for cross-modal and modality-specific feature contexts to investigate whether integrating multimodal semantic content, that is content, which is represented in distributed semantic networks, incurs a processing cost. Specifically, we hypothesize that integrating features represented within a single modality-specific network is faster than integrating features across modalities. In Experiment 2, we used EEG to measure changes in oscillatory neuronal activity during the target word when participants were asked to integrate features from the same or different modalities. Oscillatory neuronal activity could be a neural mechanism that contributes to semantic integration by linking modality-specific networks to multimodal convergence zones such as ATL. In line with this idea, we hypothesize that integrating semantic information from multiple modalities will be reflected in enhanced low frequency oscillatory activity in multimodal convergence zones, as well as substantial network interaction between these regions and a widespread cortical network.

## Experiment 1

In Experiment 1 participants indicate whether two features (e.g., *silver, loud*) are consistent with a target word (e.g., *WHISTLE*). Specifically, a feature pair could either be associated with modality-specific or cross-modal semantic content. We hypothesize that integrating modality-specific feature pairs is faster than integrating cross-modal feature pairs, highlighting that word meaning is integrated more readily within modality-specific semantic networks than across.

### Methods

#### Participants

Sixteen healthy individuals participated in Experiment 1 (13 female), all of which had normal or corrected to normal vision and no known auditory deficit. The age range was 18 to 24 (*M* = 19.88).

All participants were students at the University of York, and participated on a voluntary basis. As compensation for their participation, participants received either a financial reward or course credits. Participants gave written informed consent according to the Declaration of Helsinki. In addition they were given the opportunity of a more detailed debriefing after the study. The study was approved by the Ethics Committee of the Psychology Department at the University of York.

#### Stimulus material

**Figure 1:**
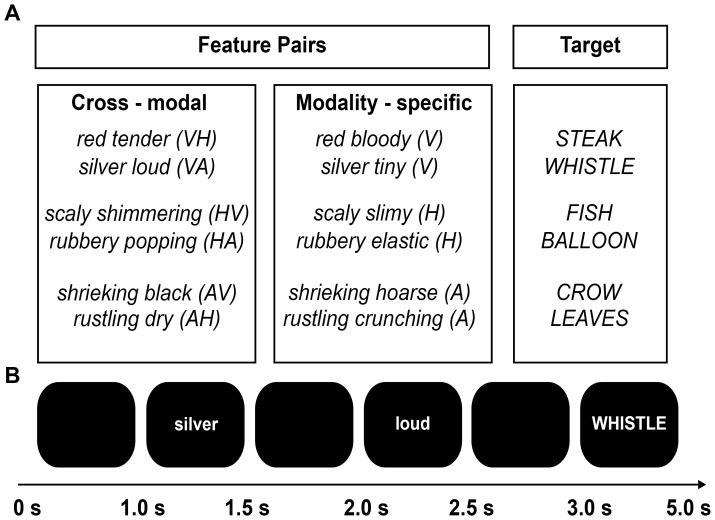
Experimental design of the dual property verification paradigm. A The top panel provides an overview of the design in which a target was either paired with a cross-modal (visual-haptic [VH; HV], visual-auditory [VA; AV], auditory-haptic [AH; HA]), or modality-specific feature pair (Visual [V], Auditory [A], Haptic [H]). The three modalities of interest were visual, haptic, and auditory. B The bottom panel depicts the time course of a single trial. All words are presented one after the other. Therefore, features can only be fully integrated when the target appears (e.g., *WHISTLE*).

120 target nouns (e.g., *WHISTLE*) were each paired with two adjective features from the same (e.g., *silver-tiny*), and two features from different modalities (e.g., *silver-loud*) (Figure 1a). Crucially, targets were presented only in one of the two feature contexts. That is, each participant saw 60 targets with a modality-specific (MS) feature pair and 60 different targets with a cross-modal (CM) feature pair. The conditions were counterbalanced and trials were presented in a pseudo-randomized order. In addition, 60 trials were included in which at least one feature was false. To familiarize participants with the experiment 10 additional practice trials were presented before the start of the experiment. Thus, each participant saw 190 target words and feature pairs.

Since the target (*WHISTLE*) and one feature (*silver*) were the same in both conditions, only variable features (*tiny, loud*) were matched for word frequency (log-scaled, British National Corpus), and length. In order to control for differences in semantic association between feature pairs and targets, latent semantic analysis (LSA) scores were extracted for each feature pair and target combination. LSA is a measure of semantic similarity that quantifies how commonly two or more words occur in the same context in written texts [28]. For example highly associated words like *camel* and *hump* yield a higher LSA score (LSA  = .53) than less highly associated words such as *camel* and *hairy* (LSA  = .20). Lastly, each feature pair was rated on a five-point scale (N  =  18) for how diagnostic and how related it is to its target word. None of these scores differed significantly between conditions (see Table 1).

**Table 1:** Matching of the experimental items.

Feature Pair	LSA	Relatedness	Diagnosticity	Frequency	Length
**Cross-modal**	0.21 (.01)	3.28 (.06)	2.65 (.07)	3.88 (.07)	6.48 (.18)
**Modality-specific**	0.22 (.01)	3.37 (.07)	2.66 (.08)	3.87 (.08)	6.24 (.17)
**p-value**	(*p* = .66)	(*p* = .32)	(*p* = .92)	(*p* = .93)	(*p* = .48)

Scores were averaged over all items in each condition. P-values were computed using independent-samples t-tests. The standard error of the mean is provided in brackets.

**Figure 2:**
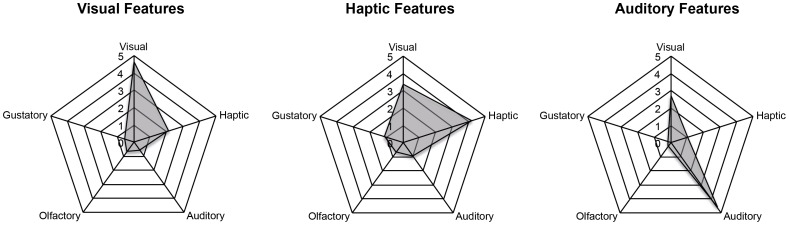
Mean of the modality ratings for visual, haptic, and auditory features. The three spider plots indicate the mean rating score [29], [30] over all features in the each of the three modalities of interest (Visual, Haptic, and Auditory).

Language is inherently polysemous, and most semantic features can be associated with multiple modalities, depending on the context. For example, a feature like *high* can be used to describe the size of a mountain (visual) or the pitch of a sound (auditory). This issue was addressed recently in two norming studies [29], [30]. Specifically, participants were asked to rate features in isolation or as feature-concept pairs on how likely the feature is experienced through one of five modalities (visual, haptic, auditory, olfactory, and gustatory). The features in the current study were based on averaged ratings from previous studies [29], [30] and a small proportion (2.6%) of additional auditory features (e.g., *ticking, quacking*). Features were selected, which had been categorized as predominantly visual, haptic, or auditory (see Figure 2).

All stimuli were presented using Neurobehavioral Systems Presentation software (www.neurobs.com) on a 22” TFT screen with a screen resolution of 1680×1050 and a refresh rate of 60 Hz.

#### Procedure

Participants were seated in front of a computer screen at a distance of 40 cm. Words were presented in light grey on a black background with a font size of 40 pt. Each trial started with the disappearance of a fixation cross that was presented at a variable interval between 1500 and 2500 ms. Individual features were presented subsequently, for 500 ms, with a 500 ms blank screen in between. The target was presented last (Figure 1b). Participants were instructed to indicate whether both features are consistent with the target. Responses were provided on a button box while the target was on the screen (2000 ms). Response times and number of errors were measured for subsequent analyses. Each participant saw a target only once and in one of two conditions (CM or MS).

### Results and Discussion

One participant was excluded from the analysis because performance rates on the task were at chance. Furthermore, outliers at three standard deviations from the mean were excluded from the analysis.

In order to test whether participants were able to perform the task, a one-sample t-test was conducted on the proportion of correctly identified feature-target pairs, against a test-value of 0.5. This test confirmed that participants' performance on the task was well above chance (*t*(14)  =  15.43, *p*<.001) with a mean proportion of .73 correctly recognized features.

To test for a main effect of modality-specificity, the median reaction time was computed for each condition and participant, and averaged separately for MS (visual, auditory, haptic) and CM (visual-auditory, auditory-haptic, and visual-haptic) feature pairs, resulting in two values per participant (CM and MS). The distribution of these values across participants met the assumptions of a paired-sample t-test. The test statistic revealed that participants were overall slower to respond to CM (*M* = 981.6, *SE* = 64.64) versus MS (*M* = 909.36, *SE* = 55.95) feature pairs (*t*(14) = 3.65, *p* = .003).

**Figure 3:**
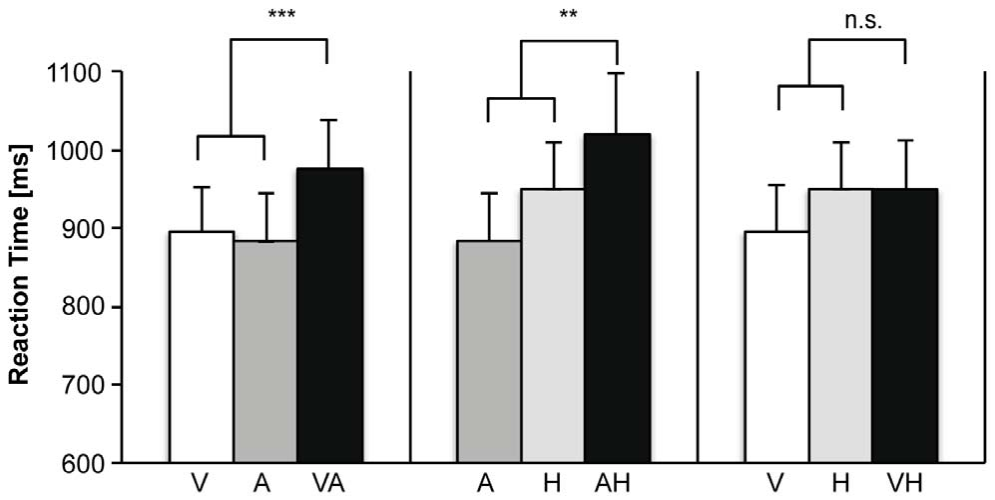
Cross-modal integration costs in verification times. Bar graphs depict the mean verification time in the MS (Visual, Auditory, and Haptic), and CM condition (Visual-Auditory, Auditory-Haptic, Visual-Haptic). Error bars denote standard error of the mean (*** *p*<.001; ** *p*<.01).

The effect of modality-specificity on verification time was further investigated for each of the three possible modality combinations using analysis of variance (ANOVA) with repeated measures (Figure 3). In each analysis, a CM condition (e.g., visual-auditory) was compared to two MS conditions (e.g., visual and auditory). The first ANOVA tested for an effect of condition on verification time across the visual (V), auditory (A), and visual-auditory (VA) conditions. The test revealed a significant main effect of condition (Wilks' Lambda  = .33, *F*(2,13) = 13.24, *p* = .001, partial η^2^ = .67). Planned comparisons using a Helmert contrast indicated that participants responded more slowly during CM (visual-auditory) than MS feature pairs (visual and auditory, respectively) (*F*(1,14) = 26.67, *p*<.001, partial η^2^ = .66). The second ANOVA tested for a main effect of condition on verification time across the auditory (A), haptic (H), and auditory-haptic (AH) conditions. The results showed a significant main effect of condition (Wilks' Lambda  = .43, *F*(2,13) = 8.61, *p* = .004, partial η^2^ = .57). Planned comparisons using a Helmert contrast revealed that participants verified CM feature pairs (auditory-visual) more slowly than MS feature pairs (auditory and haptic respectively) (*F*(1,14) = 9.22, *p* = .009, partial η^2^ = .40). The final ANOVA was conducted to test for a main effect of condition across the visual (V), haptic (H) and visual-haptic (VH) condition. There was no main effect in this analysis (Wilks Lambda  = .72, *F*(2,13) = 2.64, *p*>.1).

The goal of Experiment 1 was to investigate whether integrating semantic features represented within a single modality is faster than integrating features across modalities. The current results suggest that this is indeed the case. Verification times for two semantic features with respect to a target (e.g., *WHISTLE*) were delayed when participants saw two features from different modalities (e.g., *silver, loud*). However, this effect seems to be restricted to visual-auditory, and auditory-haptic feature combinations. A possible explanation for this finding is that visual lexical-semantic features can be difficult to distinguish from haptic features. This was also evident in the rating study in which features were often rated similarly as being experienced by seeing, and touching (Figure 2) [29], [30].

## Experiment 2

Experiment 2 uses EEG to investigate oscillatory dynamics during semantic integration within, and across different modalities. We hypothesize that integrating cross-modal semantic content will be reflected in enhanced low frequency oscillatory activity in multimodal semantic hubs, such as ATL, as well as substantial network interaction between these regions and a widespread cortical network.

### Methods

#### Participants

For Experiment 2, 22 healthy participants (8 female) were tested, all of which had normal or corrected to normal vision and no known auditory deficit. The age range was between 19 and 34 (M =  21.26). Four participants were excluded from the analysis due to excessive movement and blinking (3), and a technical error (1). None of the participants had participated in Experiment 1

Participants gave written informed consent according to the Declaration of Helsinki. In addition they were given the opportunity of a more detailed debriefing after the study. The study was approved by the Ethics Committee of the Psychology Department at the University of York.

#### Stimulus material

The stimulus materials in Experiment 2 were exactly the same as in Experiment 1.

#### Procedure

In Experiment 2, participants were wearing an electrode cap that was connected via an amplifier to the recording computer while performing the verification task. The setting was the same as in Experiment 1. However, in order to prevent contamination of the EEG signal from movement and response planning [31], the task was changed such that participants only responded in case they encountered a false feature.

#### Data recording and pre-processing

EEG was acquired from 64 Ag-AgCl electrodes that were positioned on an electrode cap according to a 10–20 system. All electrodes were re-referenced offline to the algebraic average of the two mastoids. Horizontal and Vertical eye movements were recorded with a set of bipolar Ag-AgCl electrodes. The signal was amplified using an ANT amplifier with a band-pass filter between 0.5 and 100 Hz. Impedances of the cortical electrodes were kept below 10 kΩ. The signal was recorded with a sampling frequency of 500 Hz.

Offline analyses were conducted using Matlab 7.14 (Mathworks, Natick, MA) and Fieldtrip, a Matlab toolbox for analyzing EEG/MEG data [32]. Trials were only considered if the participant correctly withheld the response on a target. Artifact rejection was performed in three consecutive steps. First, muscle artifacts were removed using semi-automatic artifact rejection. Subsequently, extended infomax independent component analysis (ICA), with a weight change stop criterion of <10^−7^, was performed to identify, and reject ocular components. Finally, each trial was visually inspected for any remaining artifacts. The average number of correct trials that survived the rejection protocol did not differ significantly between condition (MS: *M* = 48, *SE* = 1.26; CM: *M* = 47, *SE* = 1.24; *t*(17) = −1.29, *p*  = .21).

#### Spectral analysis

In order to estimate spectral power changes over time, time-frequency representations (TFR) were computed for each trial, using a 500 ms fixed sliding time window with time steps of 50 ms, resulting in a frequency resolution of ∼2 Hz. A Hanning taper was applied to each of these segments to reduce spectral leakage. TFR's were calculated for frequencies between 2 and 20 Hz in steps of 2 Hz. These transformations were performed at the individual trial level and reflect both evoked and induced components of the signal. Subsequently, trials were averaged for each condition and subject, and percentage signal change was computed using a common baseline over both conditions. The time window for the baseline was between 750 and 250 ms before the onset of the trial. The baseline normalization procedure is equivalent to the event-related de-synchronization technique (Pfurtscheller & Lopes da Silva, 1999), except that positive values denote synchronization, and negative values de-synchronization ((active-passive)/passive*100). Total power was averaged over 6 regions of interest.

#### Statistical analysis

Inferential statistics on the time-frequency windows following the presentation of the target word were computed using a cluster-based permutation approach [33]. Cluster-based permutation effectively reduces the number of comparisons by clustering neighboring samples above a given threshold along the dimensions: time, frequency, and space. In the current study, paired-sample t-tests were computed over subjects for each ROI-time-frequency point (0–1000 ms, 2–20 Hz, 6 ROI). Subsequently, t-values were thresholded at α = .05. Neighboring t-values above the threshold criterion were included into the same cluster, and ranked according to the size of the cluster. Finally, cluster-level statistics were computed by comparing the sum of all t-values within a given cluster against a permutation null-distribution. The null-distribution was constructed by randomly permuting the conditions (iterations = 1000), and calculating the maximum cluster-level statistic for each iteration.

A similar procedure was used for the seed-based whole-brain connectivity analysis. The difference between each condition (CM and MS) and the baseline was computed for an early (0–500 ms) and late (500–1000 ms) time window. The value at each location in source space was thresholded using a permutation distribution (α = .05, 1000 iterations), and combined with values from spatially adjacent locations. We used a maximum statistic to control for multiple comparisons at the cluster-level, which was equivalent to the sensor space analysis.

#### Source reconstruction

Sources of oscillatory activity at the whole-brain level were estimated using a linear beamforming method (Gross et al., 2001; Liljeström et al., 2005). The forward model was computed on a regular three dimensional grid (10×10×10 mm spacing) using a realistic volume-conductor model [34]. Paired-sample t-tests were computed for the difference between conditions at each location in the brain. Subsequently, t-values were transformed into z-values and masked at α  =  0.05.

#### Connectivity analysis

The analysis of cortico-cortical connectivity in source space was conducted for an early (0–500 ms) and a late time window (500–1000 ms) at the frequency that showed the strongest power difference in sensor space (∼6 Hz). The same number of trials were randomly selected for the CM and MS condition as well as the baseline period. A cross-spectral density (CSD) matrix was computed from the tapered Fourier spectra of each trial and used to estimate filter coefficients for the adaptive spatial filter. Subsequently, the Fourier spectra were projected through these filter coefficients along the strongest dipole orientation.

Functional connectivity between each location in the brain and all others was estimated using the imaginary part of coherency (ImCoh). ImCoh is only sensitive to signals at a non-zero time-lag, and therefore insensitive to connectivity artifacts resulting from volume conduction [35]. We computed ImCoh based on the Fourier spectra at each location in the grid. Subsequently, a stabilizing z-transform was applied using the inverse hyperbolic tangent (tanh^−1^). Since the main interest was in the functional connectivity between nodes rather than the direction of the effect, the absolute was computed for each of the resulting z-values.

For subsequent graph analysis, a binary adjacency matrix was computed for each participant by thresholding with the maximum value at which none of the nodes in any of the conditions was disconnected to the rest of the network. Finally, the log10 transformed difference between the number of connections (degrees) in the seed region versus baseline was computed for each condition, and subjected to statistical testing.

### Results and Discussion

**Figure 4:**
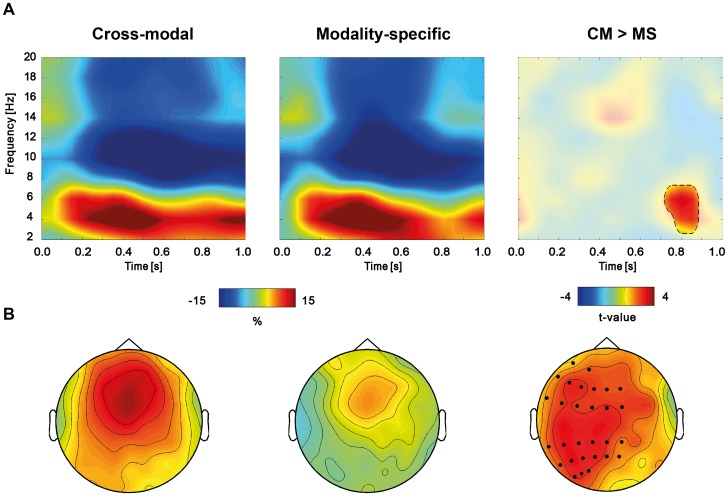
Modulation in low frequency cortical oscillations for the target word in a cross-modal or modality-specific context. A The top panel shows time-frequency representations, averaged over all significant clusters. The first two panels show the grand average percent signal change with respect to the baseline. The third panel depicts the masked statistical difference between the two conditions in t-values. The contour plot reveals one significant cluster in the theta range (4–6 Hz). B The first two bottom panels depict the topography of the effect in each condition (4–6 Hz, peak at 750–850 ms) relative to baseline. The third panel signifies the statistical difference between conditions in t-values. Electrodes within significant clusters are marked with dots (p = .002, cluster-corrected)

**Figure 5:**
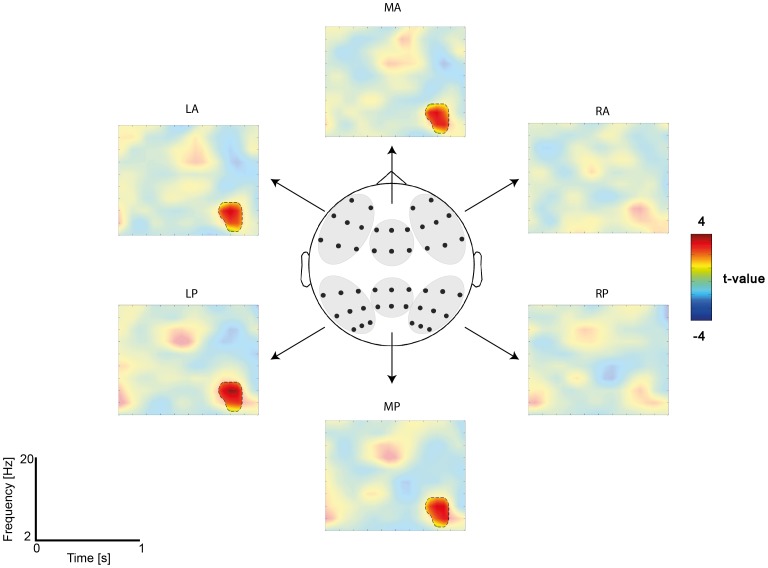
Time-frequency plots for each of the 6 ROI. The ROI were middle anterior (MA), left anterior (LA), right anterior (RA), middle posterior (MP), left posterior (LP), and right posterior (RP) electrodes. Time-frequency representations depict the statistical difference in t-values for the target word in the CM versus MS feature context. The contours indicate the peak of the cluster-corrected statistical difference (p = .002).

**Figure 6:**
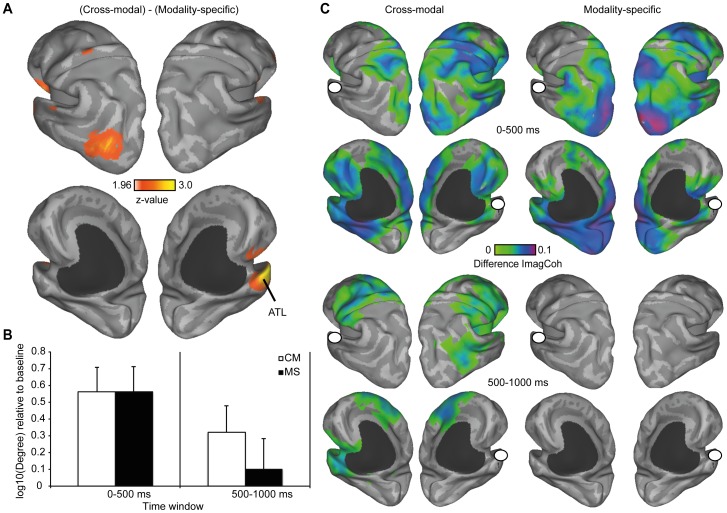
Source reconstruction and connectivity analysis. A Source reconstruction of the effect in the theta band, depicted as thresholded z-values, reveals peaks in left ATL and MOG. B Bar graphs show a significant increase in the number of connections between ATL and the rest of the brain in the early time window (0–500 ms). In the late time window (500–1000 ms), only the CM condition shows a significant increase in the number of connections relative to baseline. Error bars depict SEM C Results of the whole-brain connectivity analysis, seeded in the ATL (white dot). Connectivity maps show the difference in absolute, z-transformed, imaginary coherence between each condition and the baseline. In the early time window both conditions show a strong increase in connectivity between the ATL and a widespread cortical network. In the second time window, only the cross-modal condition shows continuing network activity above baseline.

The time-frequency analysis of total power revealed a sustained increase in the theta band (4–6 Hz) and a decrease in the alpha, and low beta band (8–20 Hz) while the target word (e.g., *WHISTLE*) was on the screen (Figure 4a). In order to test for differences between conditions (CM>MS), a cluster-based permutation approach was used [33]. In the first step of the analysis, the clustering algorithm revealed one significant cluster (4–6 Hz, peak at 750–850 ms) at left and central electrodes (LA, LP, MA, MP) (Figure 4b; Figure 5). In order to control for multiple comparisons, a maximum permutation statistic was used in which the summed cluster t-value was compared against a permutation distribution with 1000 iterations. The maximum statistic revealed a significant difference between conditions at the cluster level (*p* = .002, two-tailed), suggesting enhanced theta power in the cross-modal condition. Source reconstruction of this effect revealed a major peak in left ATL as well as left middle occipital gyrus (MOG) (Figure 6A).

The grid point in the left ATL (mni coordinate: −49 22 −30), which was most sensitive to the power difference between conditions, was taken as the seed for subsequent connectivity analyses. One sample t-tests were used to test for an increase in the log-transformed number of connections (degrees) relative to baseline in an early (0–500 ms) and late (500–1000 ms) time window. In the early time window, both conditions showed a significant increase in the number of connections (CM: *t*(17)  = .389, *p*<.001; MS: *t*(17)  = .355, *p* = .001, one-sided). However, in the late time window, an effect was found only in the CM condition (CM: *t*(17)  =  2.13, *p* = .024; MS: *t*(17) = .56, *p* = .291, one-sided). Further, paired-sample t-tests were used to test for a difference between conditions directly. A difference between conditions was observed only in the late (*t*(17) = 2.36, *p* = .031, two-sided), but not the early time window (*t*(17) = .012, *p* = .991, two-sided). Taken together, this suggests that during the first 500 ms after target onset, the ATL is communicating with a wide cortical network in both conditions (CM and MS). However, during the second 500 ms, this effect persists only in the CM condition (Figure 6B).

To illustrate which specific regions show enhanced functional connectivity with the ATL, we used a whole-brain cluster-based permutation procedure on the z-transformed ImCoh values, comparing each condition to the baseline. This approach was similar to the procedure we used in sensor-space. As depicted in the top panel of figure 6C, a large cluster of nodes was connected to the ATL in the early time window for both conditions (CM: *p* = .004; MS, *p* = .008, one-sided). However, in the late time window a significant difference relative to baseline was only observed in the CM condition (*p* = .032, one-sided). Connections during the second time window were found to regions that are involved in auditory (right Heschl's gyrus), somatosensory (bilateral post-central gyrus), and visual object processing (right posterior MTG), as well as medial and lateral frontal lobes.

The aim of Experiment 2 was to investigate whether integrating semantic features over a wider cortical network is reflected in enhanced oscillatory activity at low frequencies. Time-frequency analysis revealed an increase in theta power (4–6 Hz) for both conditions, which was more sustained during cross-modal integration. This effect localized most strongly to the left ATL, which is thought to be a major hub for integrating multimodal semantic content [18]. Subsequent seed-based whole-brain connectivity analysis confirmed that the number of connections between the ATL and the rest of the network increases in both CM and MS conditions during the first 500 ms. However, these network interactions extend into the second 500 ms only in the CM condition. Specifically, the ATL communicates with modality-specific auditory, somatosensory and high-level visual areas as well as regions in the frontal lobe. Taken together, these findings suggest that theta oscillations are associated with network dynamics in a widespread cortical network. Previous research has associated theta oscillations with lexical-semantic processing [22], [23]. However, the current study is the first to show that theta power is sensitive to the spatial distribution of semantic features in the cortex. The implications of these findings for semantic processing are discussed in the next section.

## General Discussion

Embodied theories of language have argued that word meaning is partially stored in modality-specific cortical networks, converging in multisensory association areas in anterior temporal, and inferior parietal lobes [1]–[4], [17]. The aim of the current study was to investigate the mechanisms underlying integration of semantic features during language processing. Two experiments are reported in which participants were asked to verify whether two features from the same (e.g., *silver - tiny*) or different modality (e.g., *silver - loud*) are consistent with a given target word (e.g., *WHISTLE*). The results from Experiment 1 show that integrating features from the same modality is faster than integrating features from different modalities. These findings suggest that word meaning is integrated more readily within a single modality-specific network than across networks. Integrating information across networks in particular should engage multimodal convergence zones. Experiment 2 shows that integrating features from different modalities induces a sustained theta power increase in left ATL, a putative hub for semantic convergence [18]. Low frequency theta oscillations could reflect a neural mechanism by which multimodal word meaning is combined locally in temporal association cortices. However, assuming that word meaning is partially stored in distributed cortical networks, multimodal integration necessarily requires long-range communication between left ATL and the rest of the cortex. The seed-based connectivity analysis in the theta range revealed that this is indeed the case; left ATL communicates with a widespread cortical network that includes, but is not limited to, modality-specific regions. In other words, local theta power in left ATL reflects long-range communication between temporal areas and the rest of the cortex, which, according to embodied theories of semantics, is necessary for the integration of word meaning from multiple modality-specific semantic networks.

### Integrating multimodal semantic information comes at a cost

Experiment 1 shows that participants are faster to verify features of a target word (e.g., *WHISTLE*) from the same (e.g., *silver-tiny*) versus two different modalities (e.g., *silver-loud*), suggesting that word meaning converges more readily within a modality-specific semantic network than across networks. This is in line with behavioral studies that have examined switching costs during word comprehension [36] as well as dual property verification tasks [37] (but see also [38]). It is also broadly in accordance with a cognitive model proposing graded semantic convergence from modality-specific to multimodal [39].

### Theta oscillations in left ATL during multimodal semantic feature integration

The principle by which information from distributed neural populations is combined is a much-debated topic in neuroscience. It has been argued that transient networks emerge from synchronized firing of large neuronal populations, which is recorded as oscillatory activity at the scalp [40]–[42]. In humans, changes in oscillatory neuronal activity in the theta range have been observed during different stages of memory processing, as well as lexical-semantic retrieval [22]–[25], [43]–[46]. The current study extends previous findings to show that theta oscillations are particularly sensitive to the *integration* of semantic features of an object, which are thought to be partially represented in distributed, modality-specific, networks [1]–[4].

It has been argued that modality-specific semantic networks converge in multimodal association cortices [17], [18]. For example, there is compelling evidence from patients with semantic dementia suggesting that ATL is involved in semantic processing at a general level [18], [19], yet little is known about the neural dynamics within this region. Experiment 2 reports a modulation in local theta power within left ATL when participants integrate features from multiple modality-specific semantic networks. The connectivity analysis of the data from Experiment 2 further revealed that theta oscillations also participate in long-range network interactions linking left ATL with a widespread cortical network. These findings are an important step in bridging the gap between anatomy and cognition; the theta rhythm could be a neural signature reflecting transient network interactions within left ATL, as well as between this region and distributed modality-specific networks. Such functional networks are necessary for linking semantic content in space and time.

Lastly, we find that the effect peaks at a very late point in time (∼750 ms), most likely reflecting the tail of a sustained oscillatory response that is triggered much earlier in time. Importantly, we do not argue that this is the moment when semantic integration takes place. Rather, oscillatory dynamics in the theta range could be involved in creating the conditions necessary for semantic integration by linking multiple functional networks over a period of time. The fact that cross-modal integration incurs a higher processing demand is reflected in a longer integration window. This is also in line with the finding that theta is the only known oscillatory frequency which shows a linear increase during sentence processing [47]. Again, we would like to emphasize that the primary goal of the current study was to investigate the oscillatory dynamics, rather than the timing of semantic integration, which has been addressed extensively in previous work using the event-related potential technique [48].

### Relation to multisensory integration and cross-modal matching

Multisensory integration is an essential component of everyday life. For example, both visual and proprioceptive information are required when performing goal-directed actions [49], speech comprehension greatly benefits from visual information about lip movements [50], and hearing the sound of an animal facilitates its visual detection [14]. Although these examples bear a superficial resemblance to the processes we investigated in the current study, it should be noted that there are fundamental differences in integrating cross-modal sensory, and lexical-semantic content respectively. These differences are with respect to a) the time scale and b) the directionality of information flow.

Previous studies have investigated oscillatory changes during multisensory integration using cross-modal matching. For example, Schneider and colleagues [13] showed that matching the visual image of an object (e.g., picture of a sheep) to its sound (e.g. sound of a sheep) induces an early increase in the gamma band (40–50 Hz) between 120–180 ms. Similar findings have been reported for haptic to auditory matching [15]. In contrast, effects of semantic integration in language are usually observed around 400 ms [48] and at frequencies below 30 Hz [22], [23], [51], [52] (however, see [53]). This is not surprising given the fact that lexical retrieval involves multiple processing stages (e.g., visual processing of letters). In this respect, the current findings should primarily be interpreted as reflecting language but not sensory processing.

Another difference between sensory and semantic integration is the directionality of information flow. While sensory processing in a given modality is largely automatic and dependent on external stimulation (bottom-up), retrieving modality-specific word meaning requires prior experience with the referent of a word and is highly context-dependent (top-down). For example, previous imaging work has shown that action words do not activate the action system to the same extent if they are presented as idiomatic expressions (e.g., *he kicked the bucket*) [54] (but see [55]). Furthermore, it has been shown that neutral sentences (e.g., *it is hot in here*) activate parts of the action system if presented in a context in which they are interpreted as indirect requests (e.g., a room with a closed window) [56]. In the current study, participants were primed to think about a particular instance of an object (e.g., *a silver and loud whistle*). In other words, the relevant information was not directly encoded in the stimulus (a visual word), but needed to be retrieved from memory.

In sum, imaging studies have shown that lexical-semantic content activates modality-specific cortical networks similar to sensory stimulation [5], [7], [57]. But despite their spatial similarity, lexical-semantic and sensory processes operate at very different time-scales and through different computations (bottom-up versus top-down). While much is known about the mechanisms underlying multisensory integration, the current study is among the first to address how cross-modal semantic information is integrated through language.

## Conclusions

Previous research suggests that word meaning is partially stored in modality-specific cortical networks. However, little is known about the mechanisms by which distributed semantic information is combined into a coherent conceptual representation. The current study addresses exactly this question: What are the mechanisms underlying cross-modal semantic integration? Participants were asked to indicate whether two features from the same (e.g., *silver - tiny*) or different modalities (e.g., *silver - loud*) are consistent with a target word (e.g., *WHISTLE*). Experiment 1 revealed that integrating semantic features represented within a single modality is faster than integrating features across modalities. In Experiment 2, EEG recordings revealed sustained oscillatory activity in the theta range, when participants were asked to integrate features from different modalities. The effect was localized to left ATL, a putative semantic hub that is thought to be involved in linking multimodal semantic content [18]. While the importance of this region for semantic processing and integration is largely uncontested, little is known about its mechanics. The current findings are an important step towards bridging this gap between anatomy and function; oscillatory dynamics in the theta range could be a neural mechanism that is involved in establishing transient functional connections between distributed modality-specific, and multimodal semantic networks. Further evidence for this claim is the finding that theta oscillations in Experiment 2 also participate in long-range interactions linking left ATL to a widespread cortical network.
